# Data of phosphoproteomic analysis of non-functioning pituitary adenoma

**DOI:** 10.1016/j.dib.2018.03.085

**Published:** 2018-03-27

**Authors:** Ashutosh Rai, B.D. Radotra, K.K. Mukherjee, S.K. Gupta, Pinaki Dutta

**Affiliations:** a Department of Neurosurgery, Postgraduate Institute of Medical Education and Research, Chandigarh 160012, India; b Histopatholgy, Postgraduate Institute of Medical Education and Research, Chandigarh 160012, India; c Endocrinology, Postgraduate Institute of Medical Education and Research, Chandigarh 160012, India

## Abstract

Here we describe data of a comprehensive phosphoproteomic evaluation of 20 non-functioning pituitary adenomas (NFPAs). Peptides from 20 tumor samples were enriched with TiO_2_ beads and fractioned using bRPLC and subjected to high throughput LC-MS/MS-Orbitrap Fusion™ Tribrid™ Mass Spectrometer for analysis. Upto 5 precursor ions were selected for MS/MS analysis. Data was analyzed using MASCOT and SEQUEST. Bioinformatics tools Phosphosite Plus, Gene Ontology, DAVID, and KEGG were used to determine the biological significance of identified phosphoproteins.

In this study, 2508 phosphopeptides corresponding to 1345 phosphoprotein were identified. The phospho EGFR, MEK, and STAT1/3, β-Catenin, BRAF, and HSPB1 were significantly hyperphosphorylated in the recurrent group as compared to the non-recurrent NFPA.

Identification of these phosphoproteins provides a roadmap for patient stratification, prognostication for recurrence and trials for targeted therapy.

**Specifications table**

**Table:** 

Subject area	Biology
More specific subject area	Pituitary tumor phosphoproteomics
Type of data	Excel Tables
How data was acquired	Orbitrap Fusion Tribrid mass spectrometer (Thermo scientific, Bermen, Germany)
Data format	Analyzed output data
Experimental factors	TiO_2_ enrichment of phosphoproteins from NFPAs
Experimental features	Quantitative (TMT-labeling) phosphoproteomic analysis of NFPAs
Data source location	Postgraduate Institute of Medical Education and Research, Chandigarh, India
Data accessibility	Supplementary Table 1

**Value of the data**

• To the best of our knowledge this dataset is first global phosphoproteomic analysis of non-functioning pituitary adenomas
• Data provides comparative and quantitative expression of phosphoproteins in invasive and recurrent as compared to non-invasive and non-recurrent NFPAs.
• This dataset is a useful resource of differentially expressed phosphoproteins that can serve as prognostic and therapeutic marker of NFPAs

## 1 Data

**Fig. 1:**
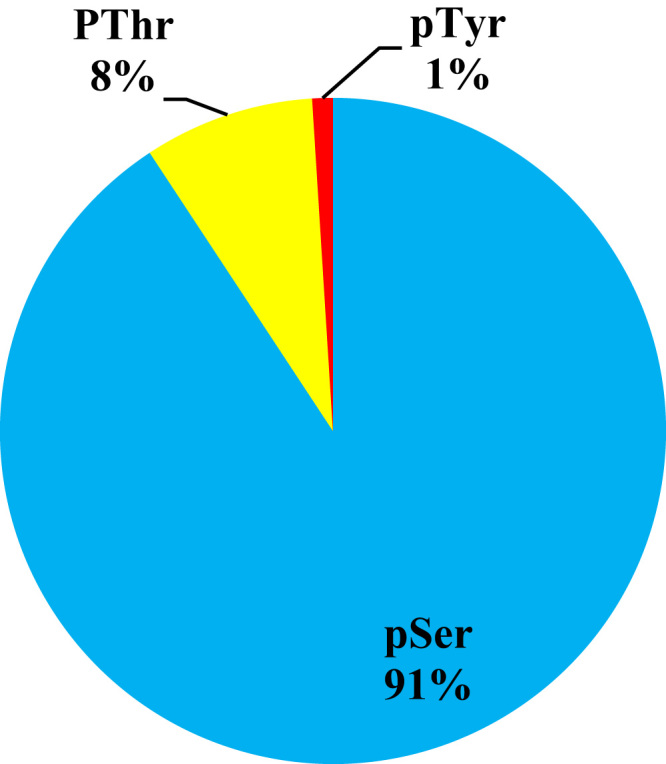
Distribution of pSerine, pThreonine and pTyrosine containing peptides.

**Fig. 2:**
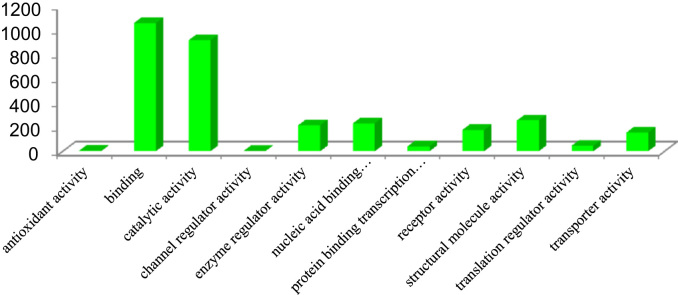
Molecular functions based on GO classification.

**Fig. 3:**
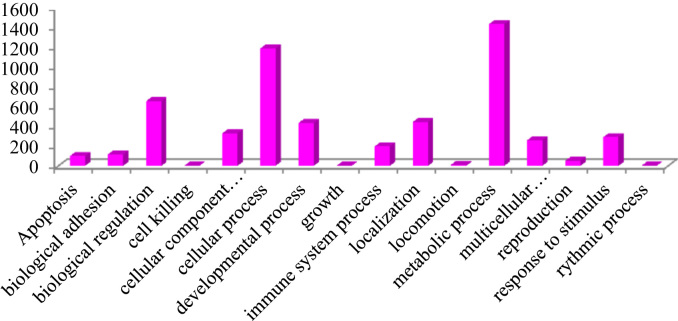
Biological process based on GO classification.

**Fig. 4:**
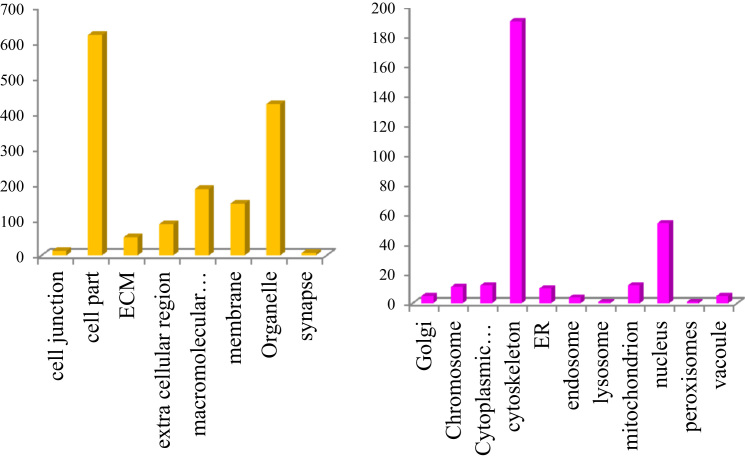
Cellular localisation of identified phosphoproteins based on GO classification.

**Fig. 5:**
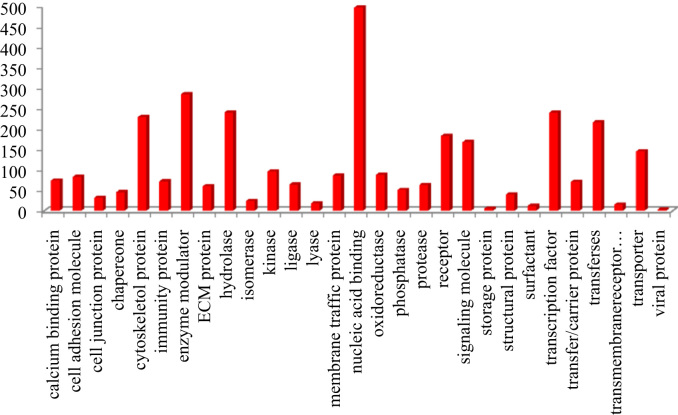
Protein classes based on GO analysis.

**Fig. 6:**
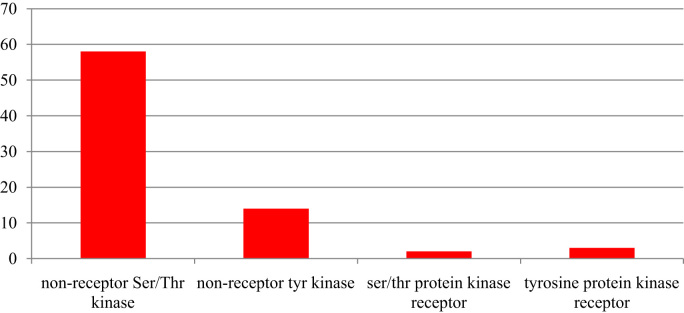
Distribution of kinases of identified phosphoproteins based on GO classification.

This dataset contains raw and processed data of LC-MS/MS analysis expressed phosphoproteins of non-functioning pituitary adenomas. In this study, 1345 phosphoprotein groups and 2233 unique phosphopeptides were identified. (Supplementary table) Tables 1 summarize the results obtained from this analysis. We identified 2157 phosphopeptides with phosphorylation at serine residues, 198 phosphopeptides with threonine phosphorylation, and 24 tyrosine-phosphorylated peptides (Fig. 1). Using the bioinformatics pipeline (PhosphositePlus, Gene Ontology, DAVID, and KEGG) molecular functions to these phosphoproteins involved in cell proliferation and growth (Fig. 2, Fig. 3, Fig. 4, Fig. 5, Fig. 6) were identified which can serve as potential prognostic markers. Dysregulated phosphoproteins from this data can be utilized for validation on large cohort of NFPA patients. Importantly, this data can be utilized to find out potential therapeutic targets for which already FDA-approved drugs are available.

## 2 Experimental design, materials and methods

### 2.1 Sample preparation

Tumors from twenty male patients of NFPAs have been collected and protein was extracted using lysis buffer 2%SDS, 1 mM sodiumorthovanadate, 2.5 mM sodium pyrophosphate, 1 mM β-glycerophosphate, protease inhibitor cocktail (Roche, Cat.No.11836153001), sonicated and centrifuged at 16,000g at 15 °C for 20 min. To measure the protein amount in lysates, Bicinchoninic acid assay (Pierce, Waltham, MA Cat #23225) was used. A series of dilutions (100 ug/ml to 2000 ug/ml) of known concentration were prepared from the Bovine Serum Albumin to make the standard curve. Pipetted out 25 ul of each standard and samples (5times diluted) into a 96 well microplate. Added 200 ul of BCA reagent and mixed thoroughly on the shaker. Covered the plate and incubated at 37 °C for 30 min. Measured the absorbance at 562 nm.

### 2.2 Digestion of proteins

Filter aided sample preparation (FASP) [1] was done to reduce the SDS amount. Amicon (Millipore) 30 kDa cut-off filters were used to buffer exchange the sample. The required amount of protein lysate (1- 1.2 mg for each channel of TMT for TiO_2_ enrichment) was taken and added DTT to a final concentration of 5 mM and incubated the sample at 60 °C for 20 min. Cooled the tubes by keeping them at room temperature. Buffer exchange was done with 8 M Urea to remove SDS by urea buffer. Added 500 µl of sample and 14 ml of 8 M Urea to 30 KDa MWCO filter and mixed well. Centrifuged the tubes at 2500 g for 20 min at 15 °C and Added IAA to a final concentration of 20 mM and incubate in dark for 10 min. This was repeated with 8 M urea to bring down the SDS concentration to 0.001% (2 to 3 times, 14 ml each time). Removed Urea by buffer exchanging with 14 ml of 50 mM TEABC 40 fold dilution so 4 times such that final urea concentration became 1 nano mole and it doesn't interfere with tandem mass tags (TMT). Estimated protein amount by BCA method. Added 0.03% SDS final concentration to the retentate (to avoid protein aggregation) before trypsin digestion. Took pre-digest (20 mg) and added Trypsin 1:20 (Enzyme to substrate ratio) directly onto the tubes and incubate the tubes at 37 °C for 12–16 h. After incubation removed the supernatant and centrifuged the tubes at 10,000 g for 5 min and added the flow through to the digest. Took post digest 20 µg and checked the digestion efficiency by running a small SDS-PAGE gel. After the confirmation of digestion efficiency preceded for TMT labeling. Dried the samples using speedvac.

### 2.3 TMT labeling and peptide fractionation

Peptides were labeled with Tandem Mass Tags and bRPLC was performed for 8 mg equivalent peptides on XBridge C18, column (Waters corporation, Milford, MA) with a flow rate of 1 mL/min using an Agilent 1200 series HPLC system containing a binary pump, auto sampler, UV detector and a fraction collector.

Each fraction was subjected to TiO_2_-based phosphopeptide enrichment. For phosphopeptide enrichment, the TiO_2_ beads (Titansphere, GL Sciences Inc.) were incubated with DHB solution (80% ACN, 1% TFA, and 5% 2, 5-dihydroxybenzoic acid) for 15 min at room temperature. Each fraction was resuspended in 5% DHB solution and incubated with TiO_2_ beads at 1:1 ratio for 30 min at room temperature. Phosphopeptide-bound TiO_2_ beads were washed three times with DHB solution and twice with 40% ACN. Peptides were eluted three times with 40 μL of 2% ammonia solution into tubes containing 10 μL of 20% TFA on ice. The peptides were dried and resuspended in 30 µL of 0.1% TFA and desalted using C18 StageTips. The eluted peptides were subjected to LC-MS/MS analysis [2].

### 2.4 Mass spectrometry analysis

Twelve fractions of enriched phosphopeptides were analyzed on Orbitrap Fusion Tribrid mass spectrometer (Thermo Electron, Bremen, Germany) interfaced with Easy-nLC II nanoflow liquid chromatography system (Thermo Scientific, Odense, Denmark). Peptides were resolved on an analytical column (75 μm × 25 cm) at a flow rate of 300 nL/min using a linear gradient of 10–35% solvent B (0.1% formic acid in 100% acetonitrile) over 90 min. The total run time including sample loading and column reconditioning was 120 min. Data dependent 34 acquisition with full scans in 350–1700 m/z range was carried out using an Orbitrap mass analyzer at a mass resolution of 120,000 at 400 m/z. Fifteen most intense precursor ions from a survey scan were selected for MS/MS fragmented using HCD fragmentation with 32% normalized collision energy and detected at a mass resolution of 30,000 at 400 m/z. Dynamic exclusion was set for 30 seconds with a 10 ppm mass window. Internal calibration was carried out using lock mass option (m/z 445.1200025) from ambient air.

### 2.5 Data analysis

The mass spectrometry derived data were searched using MASCOT (Version 2.2.0) and SEQUEST search algorithms using Proteome Discoverer 1.4 (Version 1.4.0.288) (Thermo Fisher Scientific, Bremen, Germany) against a humanRefSeq database. The search parameters for both algorithms included: N-terminal acetylation, oxidation of methionine, phosphorylation at serine, threonine and tyrosine (+79.966 Da) and TMT labeling (13C6) at lysine and arginine as variable modifications (6.02013 Da) and cysteine carbamidomethylation as a fixed modification. A precursor mass tolerance of 10 ppm and fragment mass tolerance of 0.05 Da was used. Trypsin was specified as protease and a maximum of two missed cleavages were allowed. The data were searched against decoy database and the false discovery rate was set to 1% at the peptide level. The probability of phosphorylation for each Ser/Thr/Tyr site on the peptide was calculated by the phosphoRS 3.1 node in the Proteome Discoverer (version 1.4, Thermo Scientific). For further analysis, we only considered phosphopeptides with > 75% localization probability. The phosphorylation sites that were identified with >75% localization probability but were assigned to different site by the search algorithm were manually corrected based on the phosphoRS localization probability for a given residue. Peptides with ratios greater than 1.5-fold were considered as up-regulated and with less than 0.5 as down regulated and used for further bioinformatics analysis.

The categorization of identified phosphorylated proteins in terms of molecular function, biological process and cellular component was carried out using Blast2GO algorithm and pathways were analyzed using KEGG, and DAVID.
