# Supplementary material for: Data of phosphoproteomic analysis of non-functioning pituitary adenoma
Source: Data Brief. 2018 Mar 27;18:781–6. doi: 10.1016/j.dib.2018.03.085 (PMC5996405; doi:10.1016/j.dib.2018.03.085)
Supplement: Supplementary file 2 — Supplementary material [file mmc2.docx]

|  | **Likely invasive/non-invasive** | **Invasive/non-invasive** | **Recurrent/non-recurrent** |
| --- | --- | --- | --- |
| **Phosphosites** | 804 | 1109 | 1165 |
| **Hyperphosphorylated sites** | 486 | 354 | 657 |
| **Hypophosphorylated sites** | 318 | 755 | 508 |
| **Proteins** | 416 | 557 | 610 |

**Table1: Summary of the number of proteins and class I phosphorylation sites that were identified and quantified in each condition**.
